# Redox-Sensitive Glyoxalase 1 Up-Regulation Is Crucial for Protecting Human Lung Cells from Gold Nanoparticles Toxicity

**DOI:** 10.3390/antiox9080697

**Published:** 2020-08-03

**Authors:** Angela Gambelunghe, Stefano Giovagnoli, Alessandro Di Michele, Simona Boncompagni, Marco Dell’Omo, Kerstin Leopold, Ivo Iavicoli, Vincenzo Nicola Talesa, Cinzia Antognelli

**Affiliations:** 1 Department of Medicine, University of Perugia, 06123 Perugia, Italy; angela.gambelunghe@unipg.it (A.G.); marco.dellomo@unipg.it (M.D.); 2 Department of Pharmaceutical Sciences, University of Perugia, 06123 Perugia, Italy; stefano.giovagnoli@unipg.it; 3 Department of Physics and Geology, University of Perugia, 06123 Perugia, Italy; alessandro.dimichele@collaboratori.unipg.it; 4 Department of Neuroscience, University G. d’ Annunzio of Chieti, Imaging and Clinical Sciences (DNICS) & Center for Advanced Studies and Technologies (CAST), 66100 Chieti, Italy; simona.boncompagni@unich.it; 5 Institute of Analytical and Bioanalytical Chemistry (IABC), Ulm University, 89081 Ulm, Germany; kerstin.leopold@uni-ulm.de; 6 Department of Public Health, Section of Occupational Medicine, University of Naples Federico II, 80131 Naples, Italy; ivo.iavicoli@unina.it; 7 Department of Experimental Medicine, University of Perugia, 06123 Perugia, Italy; vincenzo.talesa@unipg.it

**Keywords:** gold nanoparticles, glyoxalase 1, dicarbonyl stress, Nrf-2, epigenetic changes, DNA methylation, DNA hydroxymethylation, metastable phenotype

## Abstract

Gold nanoparticles (AuNPs) are considered nontoxic upon acute exposure, at least when they are equal or above 5 nm size. However, the safeguard mechanisms contributing to maintain cell viability are scarcely explored so far. Here, we investigated the cyto-protective role of Glyoxalase 1 (Glo1), a key enzyme involved in the control of deleterious dicarbonyl stress, in two human cell types of the respiratory tract, after an acute exposure to AuNPs with a main size of 5 nm. We found that the redox sensitive Nrf-2-mediated up-regulation of Glo1 was crucial to protect cells from AuNPs-induced toxicity. However, cells challenged with a pro-inflammatory/pro-oxidative insult become susceptible to the pro-apoptotic effect of AuNPs. Notably, the surviving cells undergo epigenetic changes associated with the onset of a partial epithelial to mesenchymal transition (EMT) process (metastable phenotype), driven by the increase in dicarbonyl stress, consequent to Glo1 inactivation. As a physiological respiratory epithelium is required for the normal respiratory function, the knowledge of the protective mechanisms avoiding or (when challenged) promoting its modification/damage might provide insight into the genesis, and, most importantly, prevention of potential health effects that might occur in subjects exposed to AuNPs, through targeted surveillance programs, at least under specific influencing factors.

## 1. Introduction

Over the last two decades, the employment of engineered nanomaterials (ENs), such as metal nanoparticles (NPs), increased in many productive industrial sectors [1] and in human healthcare. Consequently, the increasing diffusion of ENs-containing products as well as the growing environmental impact of hazardous NP pollutants have raised concerns for the growing risk associated with prolonged human exposure to these materials [2]. Today the potential toxicological effect of ENs is still controversial, and several important questions remain unanswered [2].

Gold nanoparticles (AuNPs) are the most exploited ENs in the biomedical field [3], where they are used as anticancer agents and medical tools for bioimaging and biosensing [4]. AuNPs are also used as dietary supplements in the food sector [3] and in many other industrial areas [2]. Although a few in vitro studies described that AuNPs can show cytotoxicity, depending on cell type, NP surface chemistry, and size [5,6], most of the in vitro and in vivo evidence supports the safety of AuNPs after acute exposures, at least when particles are equal or above 5 nm in size [7,8,9,10,11,12,13,14,15], or at concentrations close to those recorded in working environments [16]. Nevertheless, the underpinning safeguard mechanisms are poorly known and, more importantly, no information is available on the possible impairment of these protective mechanisms that would somehow hamper cell homeostasis and convert AuNPs from apparently harmless into potentially detrimental materials. In the attempt to investigate a possible cytoprotective defensive mechanism in human respiratory cells, upon acute exposure to 5 nm size AuNPs, we focused on glyoxalase 1 (Glo1) and related dicarbonyl stress that were previously demonstrated to play a crucial role in the biological responses to the effects of hazardous environmental and occupational pollutants [17,18,19]. Glo1 is the major enzyme detoxifying methylglyoxal (MG), a reactive dicarbonyl compound physiologically generated by intracellular metabolic pathways, and a potent glycating agent [20]. As such, MG reacts spontaneously with amino residues of proteins, lipids, and nucleic acids, leading to the formation of advanced glycation end products (AGEs). MG-derived dicarbonyl adducts exert complex pleiotropic effects in cells, culminating in distinct biological outcomes [21,22], including apoptosis [18,19,23,24] and cell transformation, through epithelial to mesenchymal transition (EMT) [17,25]. MG glycation of proteins is mainly directed to arginine residues, forming the hydroimidazolone MG-H1, while the major DNA adduct of MG is the imidazopurinone, MGdG [20]. The imbalance between MG generation (with AGEs accumulation) and its detoxification is defined as MG dicarbonyl stress [20]. In the present study, we found that the redox-dependent nuclear factor erythroid 2-related factor 2 (Nrf-2)-mediated Glo1 up-regulation is crucial to protect cells from AuNP-induced toxicity. Indeed, we showed that, as long as Glo1 functionality is preserved, cells are protected against the detrimental effects of AuNP. However, cells challenged by an inflammatory stimulus, become susceptible to the damaging effects of AuNPs that generate an overload of ROS and a dramatic inactivation of Glo1. Consequently, Glo1 functional depletion leads to the onset of a marked dicarbonyl stress status, through MG-H1 intracellular accumulation, which drives epigenetic changes and the onset of a “metastable” phenotype, a biological phenomenon characterized by a partial molecular epithelial to mesenchymal transition (EMT), a prelude to cell transformation, which plays a major role in a variety of diseases, including airway remodeling in asthma [26] and cancer [17].

## 2. Materials and Methods

### 2.1. Materials

Reagents included: MTT, Tiron, aminoguanidine bicarbonate (AG) and LPS (Sigma-Aldrich (Milan, Italy); Laemmli buffer (Thermo Fisher Scientific, Monza, Italy); BCA kit (Pierce, WA, USA).

### 2.2. Preparation and Characterization of Citrate-Stabilized AuNPs

Citrate-stabilized AuNPs were prepared and characterized, as previously described, with some modifications [27,28]. Details on the preparation and characterization can be found in Supplementary Information (SI). In brief, an aqueous solution of tetrachloroauric acid (HAuCl_4_; precursor) was mixed with sodium citrate solution (stabilizer). Then, freshly prepared sodium borohydride solution (NaBH_4_) was added rapidly for chemical reduction of gold (III) to elemental gold (0) nanoparticles (AuNPs). After complete reaction, the hydrosols were purified using the dialysis technique. Size distribution of AuNPs in the resulting suspensions was determined by transmission electron microscopy (TEM; ZEISS EM 10, Carl Zeiss Microscopy GmbH, Jena, Germany) revealing the formation of spherical particles with a diameter ranging from 3 to 23 nm, and a mode of distribution at 5 nm (Figure S1). These suspensions served as a stock for the preparation of dilute suspensions for cell experiments. The size of AuNPs in the corresponding dilutes was evaluated again by photocorrelation spectroscopy. In brief, stock suspensions of AuNPs were diluted with ultrapure water to a concentration equivalent to 1.6 µg/cm^2^, and analyzed at 25 °C, to determine the hydrodynamic diameter using a Nicomp 380 ZLS photocorrelator (PSS, Santa Barbara, CA, USA) equipped with a 35 mW He/Ne laser (=654 nm) and APD detector. The aggregation tendency of AuNP was determined by diluting the suspension as above in RPMI cell medium and water, and measuring the size change in comparison to the AuNP stock suspension. Particle size ranged from 2 to 21 nm, with a major distribution at 5 nm, which was in good agreement with that of the stock suspensions determined from TEM analysis. To evaluate the AuNPs tendency to aggregate upon seeding in the cell medium, the native AuNPs suspension was also compared with the AuNPs dispersed in RPMI cell incubation medium, at 0 and 4 h (Figure S2). These observations confirmed that the AuNP populations fed to cells remained of colloidal size, the largest aggregates being around 200 nm after 4 h.

### 2.3. Cell Culture and Treatments

Human bronchial (BEAS-2B) and alveolar (A549) epithelial cells were obtained from Merck Spa (Milan, Italy). Cells were cultured at the appropriate number in RPMI-1640 (Thermo Fisher Scientific, Monza, Italy), supplemented with 10% heat-inactivated fetal bovine serum (FBS, Thermo Fisher Scientific, Monza, Italy) and 1% antibiotics (penicillin-streptomycin) (Thermo Fisher Scientific, Monza, Italy), at 37 °C in a humidified incubator with 5% CO_2_ [29]. The confluent cells were treated with AuNPs at the final concentrations of 0.8 and 1.6 µg/cm^2^ for 3, 24, and 48 h. These concentrations at the indicated time-points were non-toxic or sub-toxic in preliminary experiments (dose-response curves). Since a biological effect was already obtained at 0.8 µg/cm^2^ for 3 h in both BEAS-2B and A549 cells, mechanistic studies were carried out under such experimental conditions. Controls were represented by vehicle, (NPs synthesis solvent)-treated cells, incubated for the same time-periods. Independent experiments were also carried out by pre-treating the cells with specific scavenging agents—the permeable superoxide dismutase (SOD) mimetic Tiron (4,5-dihydroxy1,3-benzenedisulfonic acid disodium salt monohydrate, Sigma-Aldrich, Milan, Italy) (5 mM for 24 h) and the MG scavenger [30] aminoguanidine, AG (1 mM for 3 h). The given concentrations of these compounds were based on the outcomes of previous works [17,19] and preliminary optimization experiments, showing no significant toxicity to cells and optimal efficacy in specific biochemical assays.

### 2.4. Cell Viability Assay

Cell viability was evaluated by the assessment of reduction of MTT, as previously described [18]. Cell survival was calculated relative to control cells, which were set to 100%. Since the NPs themselves might interfere with the assay mechanism [31], thus producing inaccurate results, cell viability was also evaluated by cell counting, which yielded comparable results (data not shown).

### 2.5. Apoptosis Detection

Apoptosis was detected by measuring the activation of caspase-3, using an enzyme-linked immunosorbent assay (ELISA) (Thermo Fisher Scientific, Monza, Italy), specific for activated human caspase-3, following the manufacturer’s instructions [24,25].

### 2.6. Inductively Coupled Plasma-Optical Emission Spectrometry (ICP-OES) Analysis of Cell Uptake

ICP-OES was performed to measure AuNPs uptake in cells, using a Varian 700-Es series spectrometer (Agilent, Milan, Italy). For this purpose, supernatants and pellets of BEAS-2B and A549 cells treated with AuNPs at 0.8 and 1.6 µg/cm^2^ were collected after 3, 24, and 48 h of incubation. Cells were extensively washed with PBS to remove possible, not internalized AuNPs, and counted by a hemocytometer for cell number normalization purposes. The pellets were then extracted, adding 0.5 mL of a concentrated 1:3 HNO3-HCl solution at room temperature and bath sonicated for 10 min. After proper digestion time, the obtained solutions were diluted 20 times with water. The obtained extracted samples and supernatants were submitted directly to ICP-OES analysis. The equipment was calibrated with 1M HCl standard Au solutions, in the range 0.5–15 µg/mL.

### 2.7. Transmission Electron Microscopy (TEM) Morphology Measurements

Pellets of BEAS-2B and A549 cell cultures treated with AuNP at 1.6 µg/cm^2^ for the established treatment time periods (3, 24 and 48 h) were collected and fixed with a 2.4% glutaraldehyde solution (Sigma-Aldrich, Milan, Italy). After rinsing with PBS, the pellets were stained with a 1% OsO4 solution (Sigma-Aldrich, Milan, Italy) for 1 h. After washing with PBS, the samples were dehydrated using ethanol-water solution series from 50 to 100% v/v. The dehydrated samples were then infiltrated with propylene oxide and embedded in a liquid epoxy resin. Polymerization was achieved by thermal curing in an oven at 60 °C for 72 h. After embedding, the resin block was then thin sectioned by an ultramicrotome (model), sections of 50–70 nm thickness were collected on Formva- coated copper mesh grids. Sections were viewed in a FP 505 Morgagni Series 268D electron microscope (FEI Company) equipped with Megaview III digital camera and the Soft Imaging System at 60 kV.

### 2.8. Cell Lysate and Nuclear Extracts Preparation

Total protein extraction was performed by lysing subconfluent cells with pre-cooled radio-immunoprecipitation assay (RIPA) lysis buffer (cat. 89900, ThermoFisher Scientific), enriched with Halt Protease Inhibitor Cocktail (cat. 78430, ThermoFisher Scientific) and Halt Phosphatase Inhibitor Cocktail (cat. 78420, ThermoFisher Scientific), according to the manufacturer’s instructions. Protein concentration was determined with a bicinchoninic acid (BCA) kit (cat. 23225, ThermoFisher Scientific), with bovine serum albumin as a standard. For nuclear extracts, a FractionPREP Cell Fractionation kit (Biovision, VinciBiochem, Florence, Italy) was used [19,25].

### 2.9. Glo1 Enzyme Activity Assessment

Sub-confluent cells were lysed in 100 mM KH2PO4, 1.5 mM dithiotreitol (DTT), and 1 mM ethylenediaminetetraacetic acid (EDTA) (pH 7) extraction buffer. Cell suspensions were homogenized and centrifuged at 14,000 rpm for 30 min at 4 °C. Glo1 enzyme activity was assayed by an established method [32]. In brief, the assay solution contained 0.1 mol/L sodium phosphate buffer, pH 7.2, 2 mmol/L MG, and 1 mmol/L reduced glutathione (GSH). The reaction was monitored spectrophotometrically by following the increase in absorbance at 240 nm and 25 °C. One unit of activity was defined as 1 µmol of S-D lactoylglutathione produced per minute.

### 2.10. Detection of Methylglyoxal (MG)-H1 Protein Adducts

MG-H1 protein adducts were measured by using a competitive enzyme-linked immunosorbent assay (ELISA) kit (cat. STA-811, DBA Italia S.r.l.). In brief, MG-conjugate was coated on the ELISA plate, as specified by the manufacturer. Samples or MG-BSA standards were added in duplicates to the pre-adsorbed plate. An anti-MG-specific monoclonal antibody was incubated for 1 h at room temperature, followed by washes and incubation with horseradish peroxidase (HRP)-conjugated secondary antibody, as recommended by the kit’s manufacturer. The contents of the MG-H1 adducts in the protein samples were determined through a 4P-logistic regression equation, by comparing the absorbance at 450 nm with that of the MG-BSA standard curve. A Mindray MR-96A Microplate Reader (Mindray Medical Italy S.r.l., Milan, Italy) was used for the readings.

### 2.11. Cell Transfection and siRNA-Mediated Gene Silencing

Cells were transiently transfected with ON-TARGET plus SMART pool small interfering RNA (siRNA), to Glo1 or ON-TARGET plus siCONTROL (siCtr) non-targeting pool as the negative control (all from Dharmacon RNA Technologies, Carlo Erba, Milan, Italy), using the DharmaFECT 1 transfection reagent (Dharmacon RNA Technologies, Carlo Erba, Milan, Italy), and following a standard procedure. The biochemical (enzymatic activity) and molecular (transcript level) evidence of Glo1 gene silencing was always verified. Since no significant differences were found between siCtr and untreated cells, the observed changes were compared and shown with respect to siCtr.

### 2.12. Assessment of Cellular Levels of Reactive Oxidative Species

Assessment of cellular levels of the reactive oxidative species, including levels of reactive oxygen species (ROS) and reactive nitrogen species (RNS), was performed, as previously described [21].

### 2.13. Nrf2 Activation and HO-1 Detection

Nrf2 activation was evaluated in nuclear extracts by Nrf2 Transcription Factor Assay kit (Colorimetric) (ab207223) (DBA Italia srl, Milan, Italy), following the manufacturer’s instructions. HO-1 detection was performed by a specific Human HO-1 ELISA Kit (ab133064) (DBA Italia srl, Milan, Italy).

### 2.14. RNA Isolation, Reverse Transcription, and Real-Time Reverse Transcriptase-Polymerase Chain Reaction (RT-PCR) Analyses

Total cellular RNA was isolated using TRIzol Reagent (cat. 15596026, ThermoFisher Scientific). cDNA was then synthesized from 1 µg of RNA with the RevertAid™ H Minus First Strand cDNA Synthesis Kit (cat. K1632, Thermo Fisher Scientific). Gene expression versus β-actin was evaluated by RT-PCR on a MX3000P Real-Time PCR System (Agilent Technology, Milan, Italy). The sequences of the oligonucleotide primers are reported in Table 1. PCR primers were designed using Beacon Designer 4 software (version 4.0, Agilent Technology) from published sequence data stored in the NCBI database. PCR reactions were performed in a total volume of 20 µL, which contained 25 ng of cDNA, 1X Brilliant II SYBR^®^ Green QPCR Master Mix (cat. 600828, Agilent Technology), ROX Reference Dye (cat. 600804, Agilent Technology), and 600 nM of specific primers. The thermal cycling conditions were 1 cycle at 95 °C for 5 min, followed by 45 cycles at 95 °C for 20 s and 60 °C for 30 s. In order to verify the possible co-amplification of the unspecific targets, the melting curves were performed for all primer pairs in standard conditions. The data required for carrying out a comparative analysis of gene expression were obtained by means of the 2-(∆∆CT) method [33].

### 2.15. Genomic DNA Preparation

Genomic DNA was extracted from cells by the DNeasy Blood & Tissue Kit (DBA Italia srl, Milan, Italy), according to the manufacturer’s instructions.

### 2.16. Analysis of Global DNA Methylation and Hydroxymethylation

Global genomic DNA methylation (total number of methylated cytosines in the genome) and global DNA hydroxymethylation (5-hydroxymethylcytosine: 5hmC; total number of hydroxymethylated cytosines in the genome) [34,35] in DNA isolated from BEAS-2B and A549 cells, was assayed by 5-methylcytosine DNA ELISA Kit and 5-Hydroxymethylcytosine DNA ELISA kit, respectively, following the manufacturer’s instructions.

### 2.17. Statistical Analysis

All data were generated from three independent experiments and expressed as means ± standard deviation (SD). One-way analysis of variance with Dunnett’s or Holm-Sidak’s correction was used to assess the differences among groups. Statistical significance, determined by Student’s *t*-test, was set at *p* < 0.05.

## 3. Results

### 3.1. Biological Characterization of AuNPs-Cell Interaction

**Figure 1:**
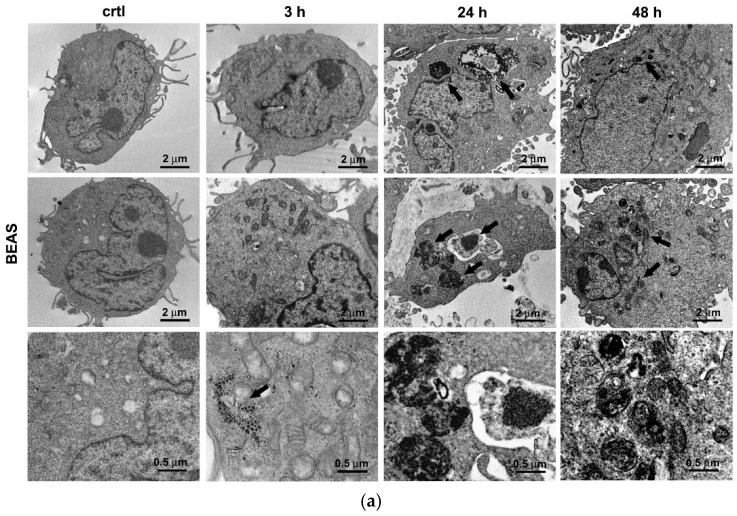

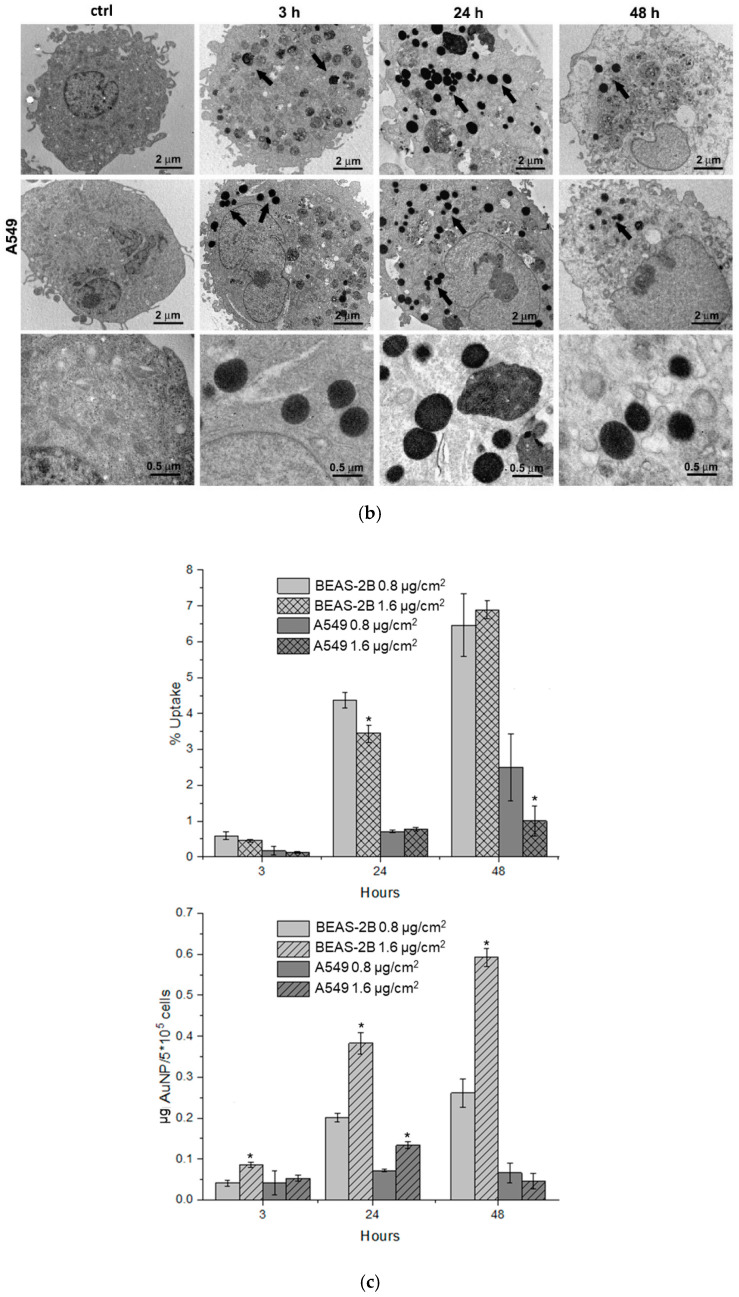
AuNPs cell interaction and uptake. Representative electron-micrographs showing AuNPs trapped inside (**a**) BEAS-2B and (**b**) A549 cells at 3, 24, and 48 h after exposure. (**a**,**b**) First column—untreated control cells (ctrl), second to fourth column—cells exposed to AuNPs for 3, 24, and 48 h, respectively. In each column, the images are sorted by increasing magnification (the first two rows: scale bar = 2 µm; the third row: scale bar = 0.5 µm). (**c**) Inductively Coupled Plasma-Optical Emission Spectrometry (ICP-OES) data showing cell uptake of AuNPs up to 48 h of incubation, expressed as the mean ± SD of the amount of Au inside cells and the percentage of Au in cells, with respect to the total Au fed to cells (*n* = 3). * *p* < 0.05, significantly different from 0.8 µg/cm^2^ in the same cell line.

TEM analysis was performed in both the BEAS-2B and A549 cells, to visualize the presence of AuNPs at 3, 24, and 48 h post-exposure (Figure 1a,b). AuNPs inside cells were visible as electron dense dark dots found mainly in the perinuclear region at all time-points. Interestingly, AuNPs appeared often in the form of either intra- or extravesicle agglomerates of random and irregular shape in BEAS-2B (Figure 1a) cells (black arrows), and were more rounded in A549 (black arrows) (Figure 1b). This was consistent with AuNPs storage in endosomal/lysosomal compartments, in membrane-bound vesicles mostly localized in the perinuclear region. Moreover, AuNPs were occasionally found to be freely dispersed in the cytoplasm and inside the nuclei, especially at shorter times of exposure (i.e., 3 h). Interestingly, TEM analysis revealed a markedly altered cell morphology, i.e., altered cell ultrastructure, uneven cell membrane, and deformation on both cell types, even if apparently more evident in A549 cells at all time-points. The ultrastructural observations were supported by a quantitative analysis by ICP-OES. In particular, we quantified the uptake of AuNPs (0.8 and 1.6 µg/cm^2^) in BEAS-2B and A549 cells at 3, 24, and 48 h post-exposure. As shown in Figure 1c, generally, a time- but not concentration-dependent uptake was observed for both cell lines. In fact, a rather similar uptake was measured at 0.8 and 1.6 µg/cm^2^ at all time-points. The only exception was for the BEAS-2B cells at 48 h that showed a more than halved uptake at the highest concentration. The uptake reached the 6–7% w/w for A549 cells and only 1–3% w/w for BEAS-2B at 48 h. This behavior was ascribed to the observed different growth profiles for the two cell lines (Figure S3), which, in particular for A549 cells, led to a dilution effect on the amount of Au internalized at later time-points. Such a hypothesis was confirmed by normalizing the Au content to cell number. In fact, an opposite behavior was observed in BEAS-2B compared to A549 cells, with a 3- to 6-fold concentration and time-dependent content increase. On the other hand, in A549 cells, the behavior was not consistent with Au content nearly independent of time and AuNP concentration.

### 3.2. Exposure to AuNPs Does Not Cause Acute Toxicity in BEAS-2B and A549 Cells

**Figure 2:**
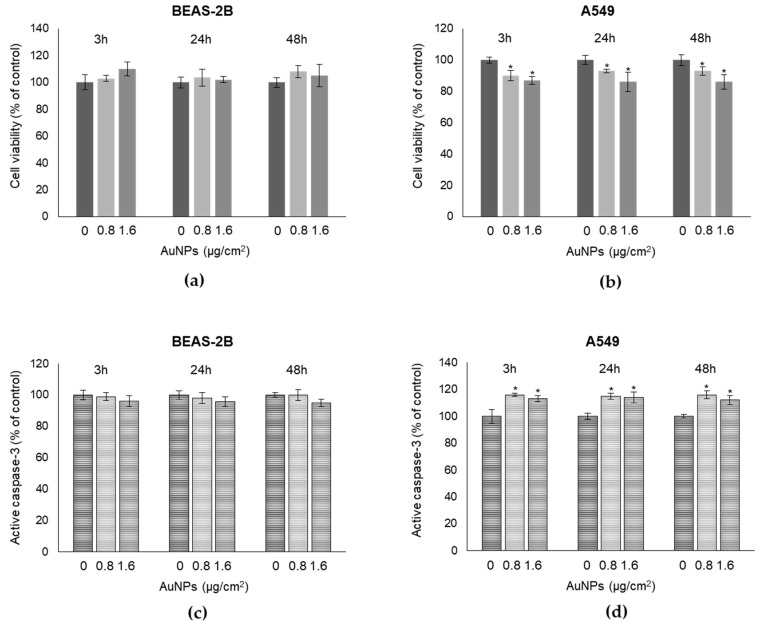
Exposure to AuNPs does not cause acute toxicity in BEAS-2B and A549 cells. Cell viability, measured by MTT assay (**a**,**b**) and apoptosis, measured by caspase-3 activation, by the specific human Caspase-3 active ELISA kit (**c**,**d**) were evaluated in human bronchial BEAS-2B and alveolar A549 cells exposed to the different indicated concentrations and post-treatment time-points of AuNPs. Data reports the means of three separate experiments performed in triplicates and the error bars represent the standard deviation (SD) of the mean. * *p* < 0.05, significantly different from the control cells.

The potential effect of AuNPs on BEAS-2B and A549 cell viability was assessed after exposure to 0.8 and 1.6 µg/cm^2^ AuNPs for 3, 24, and 48 h. We found that BEAS-2B cell viability was not affected by AuNPs at all concentrations used and time-points considered (Figure 2a). A549 cells turned out to be slightly more susceptible to AuNPs than BEAS-2B (Figure 2b). However, AuNPs exposure induced only a small decrease in A549 (10 to 13% compared to control cells), in terms of cell viability at 3 h post-exposure, which remained unchanged until 48 h (Figure 2b). Hence, all concentrations of AuNPs were in the sublethal range at all time-points analyzed. Evaluation of apoptosis by active caspase-3 detection, confirmed the results obtained from the cytotoxicity study in both cell types (Figure 2c,d). Altogether these results showed that AuNPs did not cause acute toxicity in BEAS-2B and A549 cells.

### 3.3. Exposure to AuNPs Induces Glyoxalase-1 (Glo1) Upregulation and MG-H1 Decreased Intracellular Levels in BEAS-2B and A549 Cells

**Figure 3:**
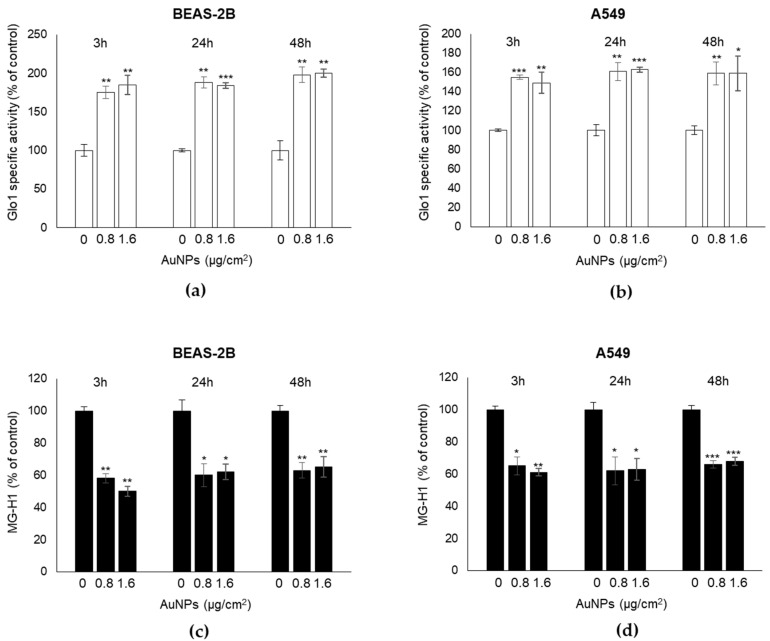
Exposure to AuNPs induces Glyoxalase-1 (Glo1) upregulation and MG-H1 decreased intracellular levels in BEAS-2B and A549 cells. Histograms show (**a**,**b**) Glo1-specific enzyme activity, measured by spectrophotometric methods, and (**c**,**d**) MG-H1 intracellular levels, assessed by ELISA. Histograms indicate means ± SD of three different cultures; each was tested in triplicates. * *p* < 0.05, ** *p* < 0.01, *** *p* < 0.001 versus unexposed cells.

In order to investigate possible mechanisms contributing to maintaining the BEAS-2B and A549 cell viability upon AuNPs exposure, we focused on Glo1, a cytoprotective enzyme whose function is to limit the intracellular accumulation of the potent pro-apoptotic agent, methylglyoxal (MG) [20]. In fact, when MG accumulates in cells at supra-physiological concentrations, it induces important post-translational modifications (PTM) that generates advanced glycation end-products (AGEs), such as hydroimidazolone (MG-H1), a heterogeneous family of compounds known to induce oxidative DNA damage and apoptosis [18,19,23,24,36]. We found that AuNPs exposure induced a significant increase of Glo1 enzyme activity, already at 3 h post-exposure, compared to the control cells in both cell lines (Figure 3a,b). The observed Glo1 increase at functional level continued at 24 and 48 h after AuNPs treatment and was concentration-independent. Accordingly, the immunodetection of MG-H1 by ELISA, performed in total protein extracts from both the control and treated cells, showed that AuNPs induced a significant decrease of MG-H1 intracellular levels (Figure 3c,d), which remained constant for the subsequent 24 and 48 h. These data suggested that the observed increase in Glo1 activity determined low physiological intracellular levels of MG-H1, which were insufficient to produce pro-apoptotic effects. As a result, the Glo1/MG-H1 axis could represent a possible protective tool preserving cell viability under AuNPs stress.

### 3.4. Glo1/MG-H1 Axis Sustains BEAS-2B and A549 Cell Viability upon AuNPs Exposure

**Figure 4:**
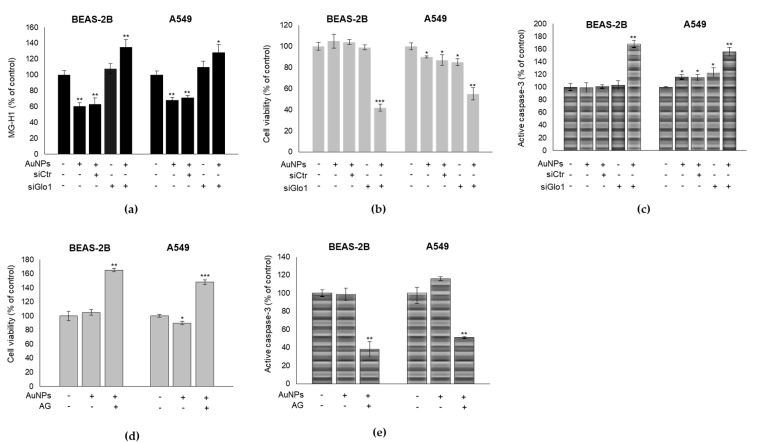
Glo1/MG-H1 axis preserves BEAS-2B and A549 cell viability upon AuNPs exposure. Effect of Glo1 silencing (siGlo1) on (**a**) MG-H1 intracellular levels, assessed by ELISA, (**b**) cell viability, assessed by MTT assay, and (**c**) apoptosis, measured by caspase-3 activation, by the specific human Caspase-3 active ELISA kit in BEAS-2B and A549 cells, treated or not with 0.8 µg/cm^2^ AuNPs for 3 h. Effect of 1 mM aminoguanidine (AG) on (**d**) cell viability and (**e**) apoptosis. Histograms indicate means ± SD of three different cultures, each one was tested in triplicates. * *p* < 0.05, ** *p* < 0.01, *** *p* < 0.001 versus unexposed cells. (−) untreated and (+) treated cells.

Glo1 silencing, successfully obtained in BEAS-2B and A549 cells at both transcriptional and functional levels (Figure S4), induced significant MG-H1 accumulation (Figure 4a), cell viability decrease (Figure 4b), and a marked increase of apoptosis (Figure 4c), compared to the AuNP-treated cells. Similarly, pre-treatment with AG, a scavenger of free MG and, consequently, of MG-derived MG-H1, resulted in a marked decrease of cell viability (Figure 4d) and increase of apoptosis (Figure 4e), compared to the AuNP-treated cells. AG treatment alone did not significantly affect cell viability (data not shown). Altogether, these results further support the possible role of the Glo1/MG-H1 axis, in preserving BEAS-2B and A549 cell viability upon AuNPs exposure.

### 3.5. The Upregulation of Glo1 and the Associated Decrease in MG-H1 Levels Caused by AuNPs Exposure Are Part of a Cell Adaptive Response to Oxidative Stress Involving the Master Redox-Sensitive Transcriptional Regulator Nrf2

**Figure 5:**
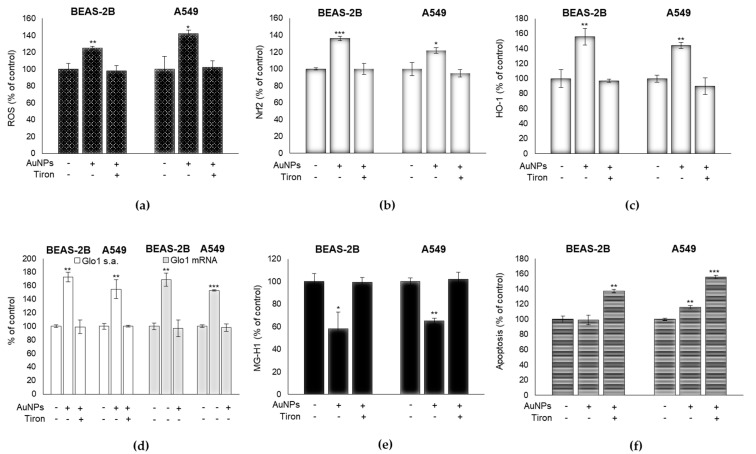
The upregulation of Glo1 and the decrease of MG-H1 levels caused by AuNPs exposure are part of a cell adaptive response to oxidative stress, involving the master redox-sensitive transcriptional regulator Nrf2. Effect of Tiron on (**a**) ROS intracellular levels, measured by H2DCF-DA assay, (**b**,**c**) Nrf2 activation assessed by a specific assay (**b**), and by HO-1 mRNA levels, detected by qRT-PCR experiments. (**c**,**d**) Glo1-specific activity and transcript levels evaluated by spectrophotometric enzymatic assay and qRT-PCR experiments, respectively, (**e**) MG-H1 intracellular levels, assessed by a specific ELISA assay, and (**f**) apoptosis, measured by caspase-3 activation, by the specific human Caspase-3 active ELISA kit in BEAS-2B and A549 cells, treated or not with 0.8 µg/cm^2^ AuNPs for 3 h. Histograms indicate means ± SD of three different cultures, each tested in triplicates. * *p* < 0.05, ** *p* < 0.01, *** *p* < 0.001 versus unexposed cells. (−) untreated and (+) treated cells.

Growing evidence reveals that Glo1 induction is involved in the molecular mechanisms underpinning cellular adaptive responses to oxidative stress under the control of the master transcription factor Nrf2 [21,22,37,38,39,40]. Hence, we tested whether the observed Glo1/MG-H1 axis could be dependent on the increase of intracellular ROS levels and Nrf2 activation. To this end, we pre-treated cells with the SOD mimetic Tiron, a mitochondria-permeable superoxide scavenger [41], at 5 mM for 24 h. After exposing cells to AuNPs, we measured Nrf2 activation by immuno-assessment of its nuclear translocation [42] and the activation of heme oxygenase-1 (HO-1), an established Nrf2-regulated enzyme, Glo1 activity at transcriptional and functional level, the MG-H1 intracellular levels, and apoptosis. As shown in Figure 5, the exposure to AuNPs caused a moderate increase of the ROS levels (Figure 5a), paralleled by Nrf2 activation (Figure 5b,c), which were both rescued by the Tiron treatment (Figure 5a–c). More importantly, the pre-incubation with Tiron significantly reduced AuNPs-induced increase of Glo1 activity and mRNA expression (Figure 5d), and increased AuNPs-induced MG-H1 depletion (Figure 5e). Finally, pre-incubation with Tiron induced a significant increase in apoptosis in both cell lines (Figure 5f). These findings indicate that the AuNP-triggered upregulation of Glo1 and the decrease of MG-H1 levels, are part of a cell adaptive response to oxidative stress, involving the master redox-sensitive transcriptional regulator Nrf2, which represents a cytoprotective mechanism for the AuNP-treated cells.

### 3.6. BEAS-2B and A549 Cells Challenged with a Pro-Inflammatory/Pro-Oxidative Stimulus Become Susceptible to the Effects of AuNPs and Show Increased ROS Production, Glo1 Inactivation, and Substantial Dicarbonyl Stress Onset

**Figure 6:**
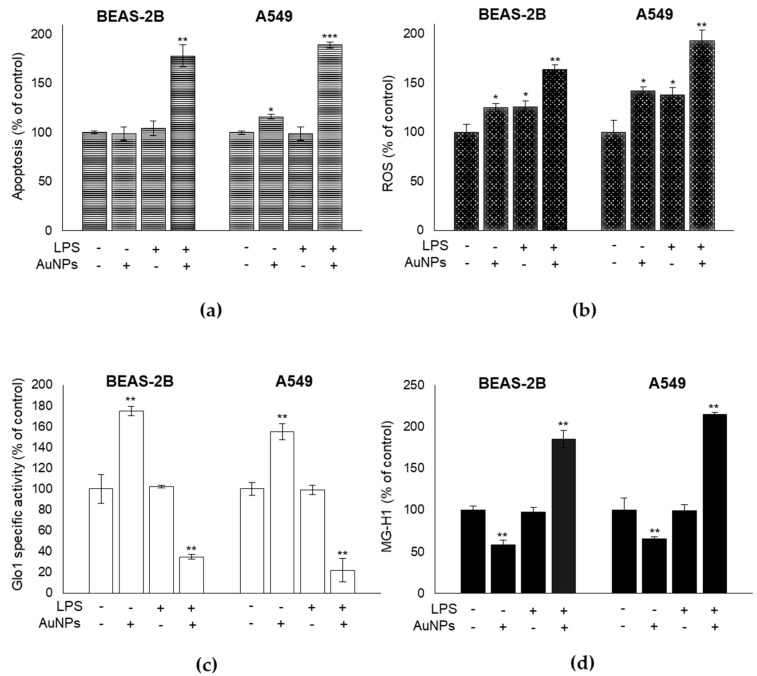
BEAS-2B and A549 cells challenged with the bacterial lipopolysaccharide (LPS) become susceptible to AuNPs effects and show increased ROS production, Glo1 inactivation, and substantial dicarbonyl stress onset. Effect of LPS (1 µg/mL for 30 min) on (**a**) apoptosis, measured by caspase-3 activation, by the specific human Caspase-3 active ELISA kit, (**b**) ROS intracellular levels, measured by H2DCF-DA assay, (**c**) Glo1 specific activity evaluated by spectrophotometric enzymatic assay, and (**d**) MG-H1 intracellular levels, assessed by a specific ELISA assay in BEAS-2B and A549 cells, treated or not with 0.8 µg/cm^2^ AuNPs for 3 h. Histograms indicate means ± SD of three different cultures, each tested in triplicates. * *p* < 0.05, ** *p* < 0.01, *** *p* < 0.001 versus unexposed cells. (−) untreated and (+) treated cells.

In order to investigate whether the AuNPs were able to exert cytotoxic effects on cells pre-challenged with a pro-inflammatory/pro-oxidative insult, we treated BEAS-2B and A549 cells with the bacterial lipopolysaccharide (LPS), previously found to induce inflammation and oxidative stress in these cell lines [43]. Once pre-treated with 1 µg/mL LPS for 30 min to induce oxidative stress and inflammation, as evident by the increase of ROS, IL-6, and IL-1β pro-inflammatory cytokines (Figure S5), the cells were seeded with 0.8 µg/cm^2^ AuNPs for 3 h. Indeed, we found a significant increase of apoptosis (Figure 6a), paralleled by a similar increase of ROS levels (Figure 6b), an imponent decrease in Glo1 specific activity (Figure 6c), and a substantial increase in MG-H1 intracellular levels (Figure 6d).

### 3.7. BEAS-2B and A549 Cells That Survived the LPS-Induced Insult underwent a “Metastable” Phenotype Associated with DNA Epigenetic Changes, through Global DNA Methylation and Hydroxymethylation, Driven by MG-H1 Accumulation

**Figure 7:**
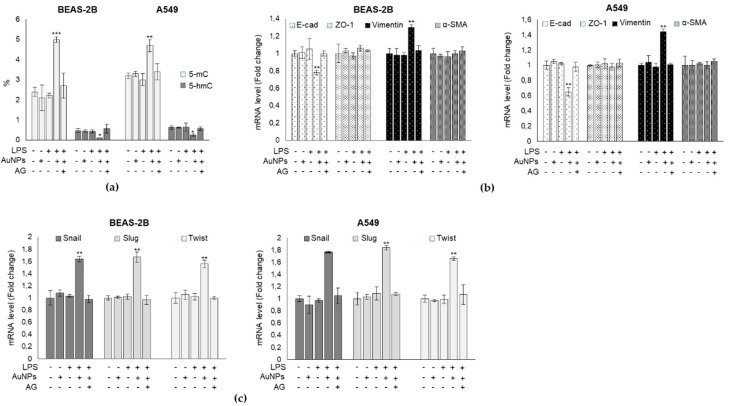
BEAS-2B and A549 cells that survived LPS-induced insults, underwent a “metastable” phenotype associated with DNA epigenetic changes, through global DNA methylation and hydroxymethylation, driven by MG-H1 accumulation. Effect of LPS (1 µg/mL), AuNPs (0.8 µg/cm^2^), and Aminoguanidine (AG, 1 mM) on (**a**) global DNA methylation (% of 5-mC) and hydroxymethylation (% of 5-hmC), measured by specific DNA ELISA kits, (**b**) transcripts levels of the intercellular epithelial adhesion molecules E-cadherin (E-cad) and zonula occludens-1 (ZO-1), and mesenchymal markers vimentin (Vim) and α smooth muscle actin (α-SMA), or (**c**) the transcription factors Snail, Slug, and Twist, evaluated by qRT-PCR. Histograms indicate means ± SD of three different cultures, each tested in triplicates. *p < 0.05, ** *p* < 0.01, *** *p* < 0.001 versus unexposed cells. (−) untreated and (+) treated cells.

It is known that MG can structurally modify DNA nucleosides through non-enzymatic glycation [44] and that several classes of environmental chemicals, including metals [45], can induce epigenetic modification (i.e., DNA methylation, histone modification, microRNAs). Notably, a recent study demonstrated that AuNPs can also alter the microRNA expression profiles in A549 cells [46]. Hence, our goal was to investigate whether BEAS-2B and A549 cells that survived LPS and AuNPs exposure and were characterized by a marked accumulation of MG-H1, the major MG-derived AGE, could undergo similar epigenetic changes. This task was carried out by focusing on global DNA methylation (total number of methylated cytosines in the genome) and hydroxymethylation (5-hydroxymethylcytosine, 5-hmC, total number of hydroxymethylated cytosines in the genome) [34,35], which, to the best of our knowledge, was never done before. Recently, 5-hmC has taken the center stage as the major DNA demethylation pathway [35]. We found that LPS-challenged cells underwent a significant increase in the global DNA methylation profile paralleled by a significant decrease in global hydroxymethylation (Figure 7a). Emerging evidence suggests that DNA methylation and hydroxymethylation variations are required for EMT [47,48,49], a multistep and progressive phenomenon, consisting of loss of the epithelial and acquisition of a mesenchymal phenotype, associated with cell transformation, playing a major role in a variety of diseases, including airway remodeling in asthma [26,50] and cancer [17,50]. Hence, we subsequently investigated whether the LPS-challenged cells exposed to AuNPs could undergo EMT and whether this transformation could be causatively linked to the observed MG-H1 accumulation. The trans-differentiation from an epithelial to a mesenchymal phenotype was accompanied by a decreased expression of intercellular epithelial adhesion molecules and induction of markers of mesenchymal cells. In order to characterize EMT, we evaluated the mRNA levels of the epithelial markers E-cadherin and ZO-1, the loss of which was considered a reliable universal marker of the disappearance of the epithelial phenotype [50], and of the mesenchymal markers, vimentin and α-SMA, which are deemed the most reliable markers of myofibroblastic cells [50]. As shown in Figure 7b, a moderate decrease was observed in E-cad mRNA expression, while that of ZO-1 remained unchanged. Similarly, a significant increase of vimentin but not of α-SMA transcript levels was observed, compared to the control cells (Figure 7b). Concomitantly, a slight increase of the mRNA levels of Snail, Slug, and Twist transcription factors, known to inhibit E-cad transcription [50], was observed (Figure 7c). AG treatment reversed both LPS/AuNPs-induced epigenetic modifications (Figure 7a) and partial EMT transformation (metastable-like phenotype) (Figure 7b,c), suggesting a causative role by MG-dependent glycative stress in triggering these processes under LPS-challenged, AuNPs exposed, BEAS-2B and A549 cells.

## 4. Discussion

A considerable body of literature recognize AuNPs as highly biocompatible and non-toxic colloidal systems [8,9,11,13,14]. Many in vitro and in vivo studies suggest that AuNPs are mostly nontoxic after acute exposures, at least as long as the particles are equal or above 5 nm in diameter [7,9,10,11,12,13,14,15]. In line with the literature, we found that 5-nm AuNPs did not cause acute toxicity in both BEAS-2B bronchial cells and A549 alveolar cells. All concentrations of AuNPs were in the sublethal range at all time-points analyzed, despite their internalization, as indicated by TEM analysis. As expected, in fact, AuNPs could easily enter and accumulate into both BEAS-2B and A549 cells. Presence of intracellular accumulated AuNPs were associated with a significant perturbation of cell ultrastructures, which was more evident in the A549 cells at all time-points. The visual observations were supported by the quantitative analysis, showing a time-dependent uptake for both cell lines.

Although 5-nm AuNPs are not cytotoxic to the BEAS-2B and A549 cells, the safeguard mechanisms underlying this cellular response are not well-known. Here, we demonstrated that the up-regulation of the anti-glycation enzyme Glo1 and the consequent decrease of cytotoxic MG-mediated dicarbonyl stress is a crucial mechanism adopted by cells to protect themselves from AuNPs-induced toxicity. Notably, these effects were shown to be redox sensitive, as they could be significantly reversed by cell treatment with the SOD mimetic Tiron, a mitochondria-permeable antioxidant, suggesting that they are part of a cell adaptive response to cope with a mild increase of intracellular ROS/RNS levels and altered redox homeostasis caused by AuNPs exposure. Accordingly, it is well established that moderately increased ROS/RNS levels might activate antioxidant response mechanisms with the aim of attenuating oxidative stress and its associated detrimental effects [21,51]. The observation that Glo1 up-regulation was ROS/RNS-dependent, extends and supports the already previously observed phenomenon of a redox-sensitive regulation of this antiglycation enzymatic defense [17,18,23,36]. Additionally, it was shown that the mammalian Glo1 gene contains a functional antioxidant-response element (ARE), which serves to engage Glo1 in the major cytoprotective antioxidant system controlled by the master redox-sensitive transcription factor Nrf2 [37], as also observed here. The activation of the cellular stress response transcription factor Nrf2 in cells exposed to AuNPs was in agreement with a very recent research in rat aortic vascular smooth muscle cells [52]. We also demonstrated that cell survival under AuNPs exposure was causatively linked, via Glo1 upregulation, to depleted MG-H1 intracellular levels, as demonstrated by Glo1 silencing experiments. In fact, under Glo1 knock-down, AuNPs led to MG-H1 accumulation and apoptosis. This was in agreement with the existing literature describing the general involvement of AGEs as apoptosis inducers and further confirmed this pro-apoptotic role to this specific MG-derived AGE [19].

As mentioned above, the cytoprotective mechanisms contributing to AuNPs toxicity response are not yet sufficiently understood. More importantly, no information is available on the possible impairment of these protective mechanisms that would somehow hamper cell homeostasis and make apparently harmless AuNPs potentially detrimental to cells. Additionally, it is well-known that humans become susceptible to NPs, including AuNPs, because of the failure to adequately respond to these exogeneous toxicants relatively well, due to a number of factors, including genetic susceptibility, tissue gene expression profile, epigenetic induced modifications, age, pathological conditions, and lifestyle factors [53]. In agreement with this, we found that cells pre-challenged with LPS and then exposed to AuNPs, were characterized by a marked increase of ROS/RNS, a dramatic Glo1 inactivation and a significant accumulation of MG-H1, paralleled by an imponent apoptosis, in agreement with the hormetic properties of ROS/RNS having antioxidant beneficial effects at low concentrations but pro-oxidant toxic effects at higher concentrations. In addition, these results corroborated the observed cytoprotective role of the ROS/Nrf2/Glo1/MG-H1 survival pathway. Since high intracellular levels of ROS can inactivate Glo1 [18,19,23,36], we hypothesized that BEAS-2B and A549 cells challenged with pro-inflammatory/pro-oxidative LPS might become susceptible to AuNPs effects through ROS-mediated Glo1 inactivation and substantial dicarbonyl stress onset, which needs further investigation.

Concomitantly, cells that survived the LPS/AuNPs insults underwent a partial molecular trans-differentiation from an epithelial to a mesenchymal phenotype. This metastable phenotype was driven by MG-H1, since AG treatment was able to efficiently prevent it. As largely known and as mentioned above, EMT transformation plays a major role in airway remodeling in asthma [26,50] and cancer [17,50]. Hence, these results seem to suggest that in pre-challenged cells, AuNPs might exert cytotoxic effects while triggering an initial transformation in the surviving cells, potentially leading to serious pathological responses through a mechanism involving the Glo1/MG-H1 axis. These data also seem to corroborate the emerging hormetic potential of MG and MG-derived AGEs in triggering different biological effects [25,54]. In fact, they drive apoptosis at high intracellular levels, whereas preserve cellular homeostasis at low concentrations. Finally, we showed for the first time that the AuNP-induced metastable phenotype in cells pre-sensitized with LPS, was associated with epigenetic changes, specifically with global DNA methylation and hydroxymethylation [34,35]. Again, AG treatment prevented both epigenetic modifications, suggesting a causative role of MG-dependent glycative stress in triggering these processes in LPS-challenged AuNPs-exposed BEAS-2B and A549 cells. Emerging evidence suggests that global DNA methylation and hydroxymethylation variations are required for EMT [47,48,49]. Since we found that MG-H1 controlled both DNA global modifications and EMT, we speculate that this MG-derived AGE can be an additional agent linking these two phenomena. How MG-H1 can induce the observed epigenetic changes remains to be explored. Since post-translational modifications by MG-derived AGEs modulate protein biological activity [55] and stability [56], we hypothesize that specific proteins inducing DNA methylation and demethylation might be activated or inactivated, respectively, by MG, leading to the observed epigenetic changes. Additionally, it was reported that AGEs can upregulate DNA methyltransferases (DNMT1, DNMT3a, DNMT3b) [57], usually modifying cytosine at position 5 in CpG dinucleotides, to create 5-methylcytosine. Alternatively, or concomitantly, a direct regulatory effect by AuNPs could not be excluded, as was observed for other enzymes [58]. This was further strengthened by the observation of AuNPs mainly in the perinuclear and nuclear regions of BEAS-2B and A549 cells.

It is known that EMT is a milestone in the carcinogenesis platform [59] and that an increase of DNA methylation and loss of global hydroxymethylation are associated with cancer [60,61]. The observation of epigenetic changes and the acquisition of an EMT-like molecular phenotype in LPS pre-sensitized cells exposed to AuNPs would possibly suggest the initiation of a carcinogenic-like transformation process, which deserve further investigation in long-term exposures.

## 5. Conclusions

In conclusion, we found that the redox sensitive Nrf-2-mediated up-regulation of Glo1, limiting intracellular accumulation of MG-derived MG-H1, is crucial to maintain BEAS-2B and A549 cell homeostasis and viability. However, when challenged with inflammatory and pro-oxidant stimuli, the cells become susceptible to AuNPs toxicity. Moreover, and notably, the surviving cells undergo epigenetic modifications associated with the onset of a metastable phenotype. As a physiological respiratory epithelium is required for the normal respiratory function, the knowledge of the protective mechanisms avoiding or (when challenged) promoting its modification/damage might provide insight into the genesis, and, most importantly, prevention (e.g., through targeted surveillance programs) of potential associated health effects that might occur in subjects exposed to AuNPs, especially workers in occupational settings, at least under specific influencing factors or predisposing conditions, such as chronic inflammatory diseases, already characterized by increased levels of detrimental AGEs.

## Figures and Tables

**Table 1:** Primer sequences.

Gene	Forward 5′-3′	Reverse 5′-3′
Glo1 (NM_006708.3)	CTCTCCAGAAAAGCTACACTTGAG	CGAGGGTCTGAATTGCCATTG
HO-1 (NM_002133.3)	AAGATTGCCCAGAAAGCCCTGGAC	AACTGTCGCCACCAGAAAGCTGAG
ZO-1 (NM_001301025.3)	GCGTCACCTACCACCTCGTCGTC	GCCTGCTGCCTTCTCCCACTCTG
E-cad (NM_001317184.2)	TTGCGGAAGTCAGTTCAG	CAGAGCCAAGAGGAGACC
Vim (NM_003380.5)	GCACACAGCAAGGCGATGG	GGAGCGAGAGTGGCAGAGG
α-SMA (NM_001141945.2)	GGCATCATCACCAACTGGGACGAC	AGCACCGCCTGGATAGCCACATAC
Snail (NM_005985.4)	GACCACTATGCCGCGCTCTT	TCGCTGTAGTTAGGCTTCCGATT
Slug (NM_003068.5)	AGCAGCTGCACTGCGATGCC	ACACAGCAGCCAGATTCCTC
Twist (NM_000474.4)	GGAGTCCGCAGTCTTACGAG	TCTGGAGGACCTGGTAGAGG
β-actin (NM_001101.5)	CACTCTTCCAGCCTTCCTTCC	ACAGCACTGTGTTGGCGTAC

## Supplementary Materials

The following are available online at https://www.mdpi.com/2076-3921/9/8/697/s1. Figure S1: Size distribution of freshly prepared AuNPs suspensions as determined by TEM. Figure S2: Particle size distribution of AuNP suspensions obtained by photo-correlation spectroscopy at room temperature. Figure S3: Growth curves of BEAS-2B and A549 cells. Figure S4: Glyoxalase 1 (Glo1) silencing by small interfering RNA (siRNA) in BEAS-2B and A549 cells. Figure S5: Induction of oxidative stress and inflammation by LPS as evident by the increase of ROS and IL-6 or IL-1β pro-inflammatory cytokines.
