# Correction: Gambelunghe et al. Redox-Sensitive Glyoxalase 1 Up-Regulation Is Crucial for Protecting Human Lung Cells from Gold Nanoparticles Toxicity. *Antioxidants* 2020, *9*, 697

**DOI:** 10.3390/antiox14050597

**Published:** 2025-05-16

**Authors:** Angela Gambelunghe, Stefano Giovagnoli, Alessandro Di Michele, Simona Boncompagni, Marco Dell’Omo, Kerstin Leopold, Ivo Iavicoli, Vincenzo Nicola Talesa, Cinzia Antognelli

**Affiliations:** 1 Department of Medicine, University of Perugia, 06123 Perugia, Italy; angela.gambelunghe@unipg.it (A.G.); marco.dellomo@unipg.it (M.D.); 2 Department of Pharmaceutical Sciences, University of Perugia, 06123 Perugia, Italy; stefano.giovagnoli@unipg.it; 3 Department of Physics and Geology, University of Perugia, 06123 Perugia, Italy; alessandro.dimichele@collaboratori.unipg.it; 4 Department of Neuroscience, University G. d’ Annunzio of Chieti, Imaging and Clinical Sciences (DNICS) & Center for Advanced Studies and Technologies (CAST), 66100 Chieti, Italy; simona.boncompagni@unich.it; 5 Institute of Analytical and Bioanalytical Chemistry (IABC), Ulm University, 89081 Ulm, Germany; kerstin.leopold@uni-ulm.de; 6 Department of Public Health, Section of Occupational Medicine, University of Naples Federico II, 80131 Naples, Italy; ivo.iavicoli@unina.it; 7 Department of Experimental Medicine, University of Perugia, 06123 Perugia, Italy; vincenzo.talesa@unipg.it

In the original publication [1], there was a mistake in Figure 1a. The corrected version of Figure 1 appears below.

The authors state that the scientific conclusions are unaffected. This correction was approved by the Academic Editor. The original publication has also been updated.

## Figures and Tables

**Figure 1:**
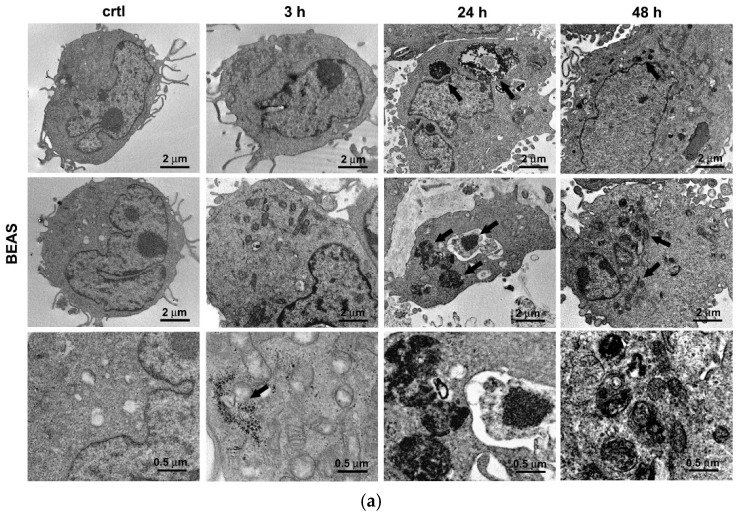

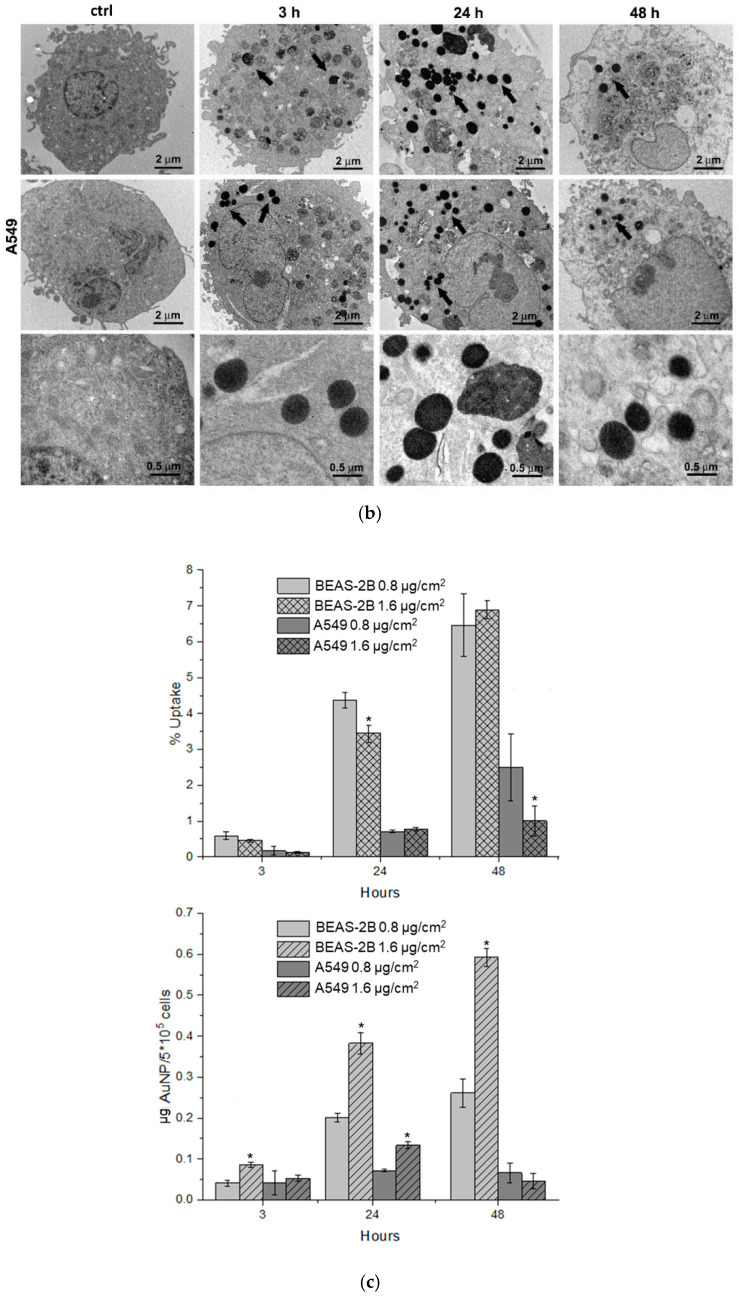
AuNPs cell interaction and uptake. Representative electron-micrographs showing AuNPs trapped inside (**a**) BEAS-2B and (**b**) A549 cells at 3, 24, and 48 h after exposure. (**a**,**b**) First column—untreated control cells (ctrl), second to fourth column—cells exposed to AuNPs for 3, 24, and 48 h, respectively. In each column, the images are sorted by increasing magnification (the first two rows: scale bar = 2 µm; the third row: scale bar = 0.5 µm). (**c**) Inductively Coupled Plasma-Optical Emission Spectrometry (ICP-OES) data showing cell uptake of AuNPs up to 48 h of incubation, expressed as the mean ± SD of the amount of Au inside cells and the percentage of Au in cells, with respect to the total Au fed to cells (*n* = 3). * *p* < 0.05, significantly different from 0.8 µg/cm^2^ in the same cell line.
