# Supplementary material for: Redox-Sensitive Glyoxalase 1 Up-Regulation Is Crucial for Protecting Human Lung Cells from Gold Nanoparticles Toxicity
Source: Antioxidants (Basel). 2020 Aug 3;9(8):697. doi: 10.3390/antiox9080697 (PMC7463694; doi:10.3390/antiox9080697)
Supplement: Supplementary file 1 [file antioxidants-09-00697-s001.docx]

**Preparation and characterization of citrate-stabilized gold nanoparticle hydrosol**

Three batches of gold nanoparticle hydrosols and two blanks (each 100 mL) were prepared. First, 0.4 mL of a fresh solution of 100 mg hydrogen tetrachloroaurate (HAuCl_4_*3H_2_O; ACS-Grade, Alfa Aesar, Germany) in 1.7 mL ultra-pure water were added to 97 g of ultrapure water. Under vigorous stirring 2mL of a citrate solution, containing 1.14 g trisodium citrate dihydrate (Na_3_C_6_H_5_O_7_*2H_2_O; p.a., Merck, Germany) in 100 mL ultrapure water, were added. Then, 1 mL of a freshly prepared 0.15 wt.-% sodium borohydride (NaBH_4_, for synthesis, Merck, Germany) solution was added, which instantly changes the color of the transparent solution from yellow to a dark red indicating reduction of Au(III) to Au(0). After 5 minutes of further stirring, the solutions were stored in the dark for 1 hour to complete the reduction reaction.

In order to remove excess of the reductant the hydrosols were dialyzed using cellulose-hydrate tubes with a mass-weight cut off of 14k (Nadir-dialysis tube, Roth, Germany). The dialysis solution, containing 1 mL of the citrate solution in 500 mL ultra-pure water, was changed after 1, 2, 4 and 8 hours. After a total of 22 hours of dialysis, the purple hydrosols were filled into glass flasks and their pH was measured (pH-electrode, Metrohm; Herisau, Switzerland).

Size distribution of ready-made hydrosols was determined by measuring at least 2000 particles per sample batch depicted by transmission electron microscopy (TEM; ZEISS EM 10, Carl Zeiss Microscopy GmbH, Jena, Germany) using ImageJ software (National Institute of Health, Bethesda, MD). The corresponding size histograms are presented in **Figure S1**. Gold concentration in the purified hydrosols were determined by total-reflection X-ray fluorescence spectroscopy (TXRF) using a S2 Picofox (BrukerNano, Berlin Gemany). Vanadium stock solution (1 g L^-1^ V in 3% HNO_3_, Fluka, Germany) was applied as internal standard and AuNP hydrosols were treated with fresh aqua regia (HCl:HNO_3_ 3:1; HCl (37% Reag. Ph. Eur, VWR); HNO_3_ (65% Normapur, VWR) for 10 seconds in order to re-dissolve gold prior to quantification.

**Figure S1**


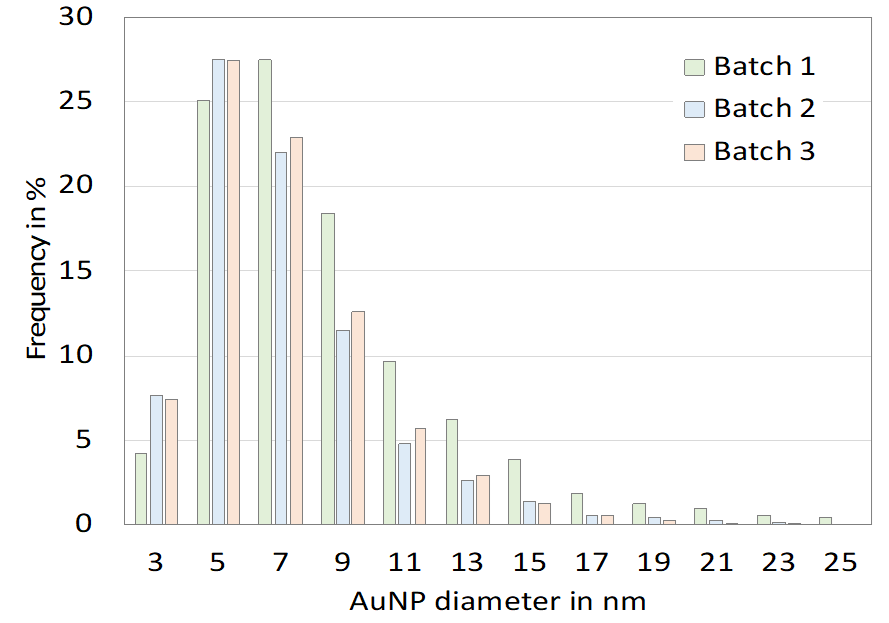


**Size distribution of freshly prepared AuNPs suspensions as determined by TEM.** The distribution was obtained by size measures of N ≥ 2,000 particle counts. Samples were prepared by direct deposition on Formvar coated copper grids and successive air drying.

**Figure S2**


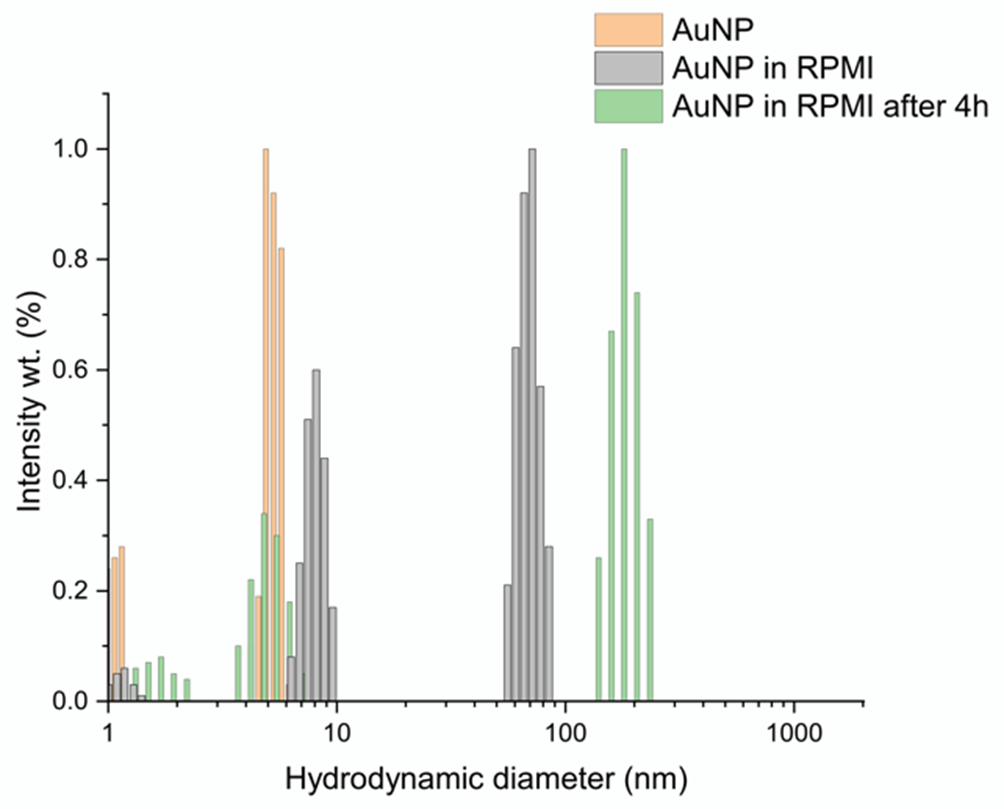


**Particle size distribution of AuNP suspensions obtained by photo-correlation spectroscopy at room temperature.** A stock AuNPs suspension was diluted in water and RPMI medium to reach the concentration equivalent to 1.6 µg/cm^2^, which is the maximum concentration used in cells. The analysis was performed over a period of 4 hr, representing an average uptake process time in cells, to estimate AuNPs aggregation tendency upon treatment.

**Figure S3**

**
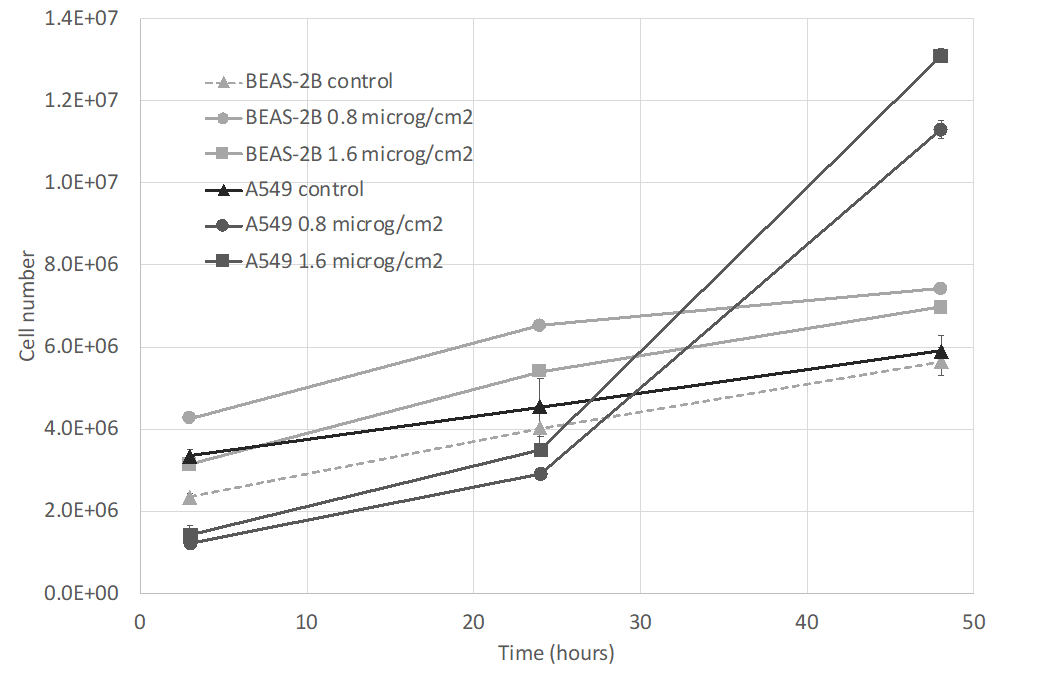
**

**Growth curve of BEAS-2B and A549 cells.** Growth curves were evaluated by cell counting. Number of cells at seeding: 1 x 10^5^ in both cell lines. Values are means ± S.D. of three experiments tested in triplicate. p < 0.01; p < 0.001 compared to cell number at seeding (0 hr). Evident the higher growth rate of A549 compared to BEAS-2B cells and the respective controls.

**Figure S4**

**
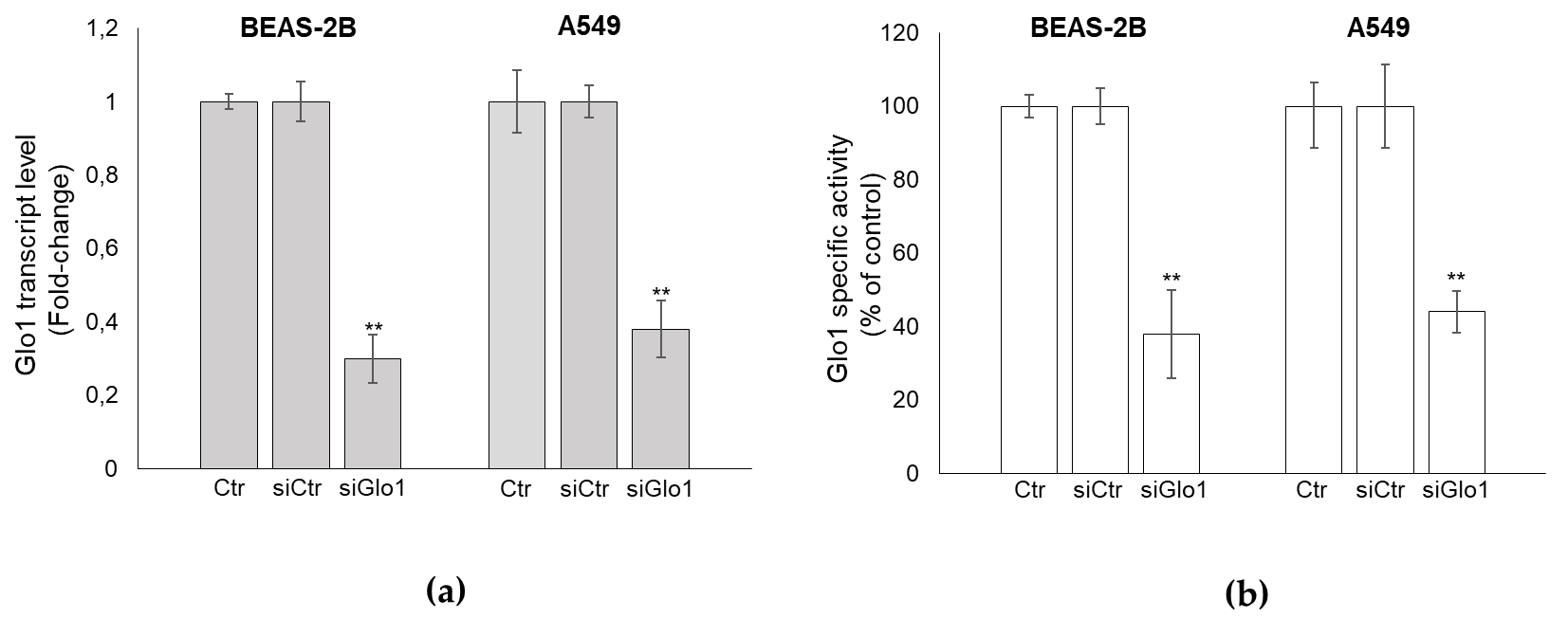
**

**Glyoxalase 1 (Glo1) silencing by small interfering RNA (siRNA) in BEAS-2B and A549 cells.** Effect of Glo1 silencing (siGlo1) on (a) Glo1 transcript level, assessed by qRT-PCR and (b) Glo1 specific activity, assessed by spectrophotometric methods. Cells were transiently transfected with ON-TARGET plus SMART pool small interfering RNA (siRNA) to Glo1 (siGlo1) or ON-TARGET plus siCONTROL (siCtr) non targeting pool as negative control following a standard procedure. Histograms indicate means ± SD of three diﬀerent cultures each of one was tested in triplicate. **p < 0.01 versus unexposed cells.

**Figure S5**

**
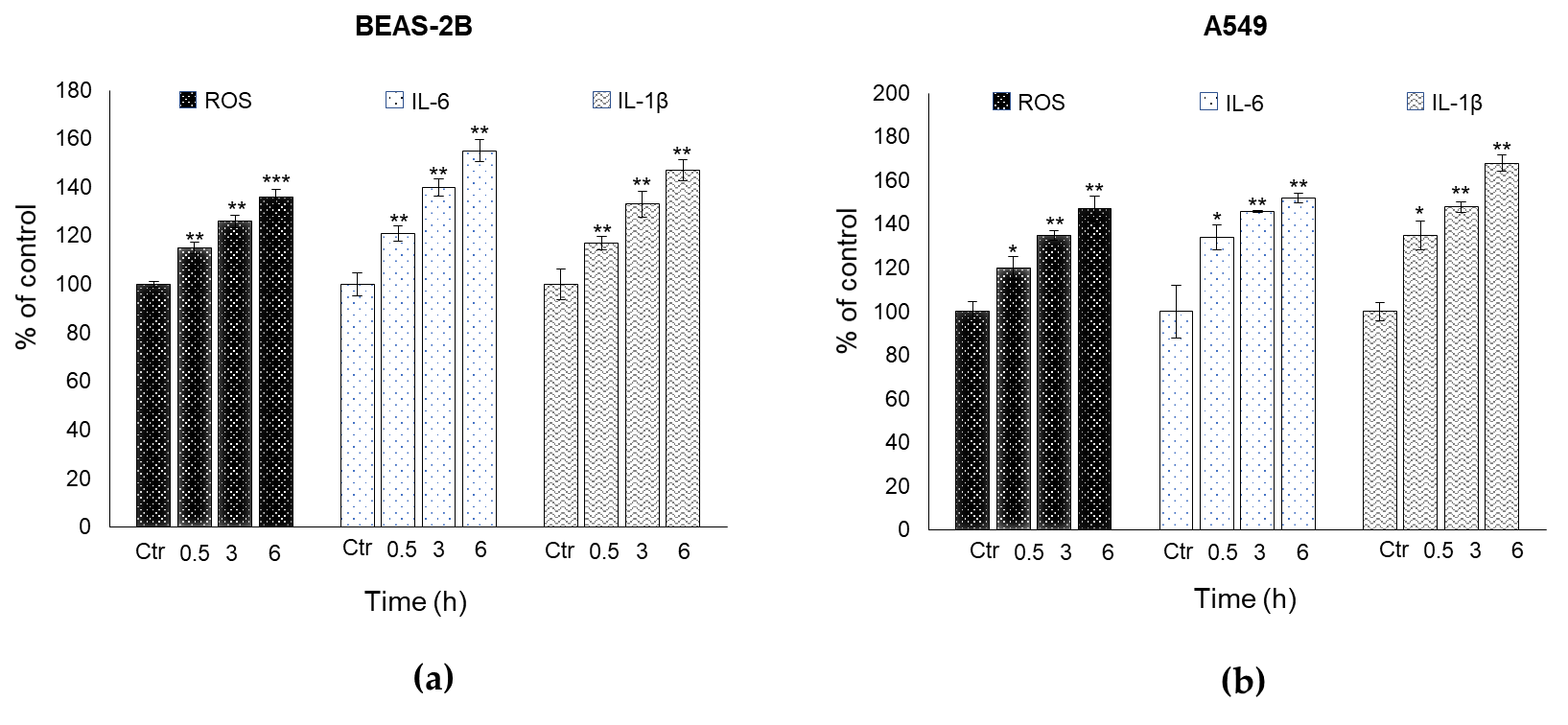
**

**Induction of oxidative stress and inflammation by LPS as evident by the increase of ROS and IL-6 or IL-1β pro-inflammatory cytokines.** (a) BEAS-2B cells and (b) A549 cells were exposed to LPS (1 µg/ml) for the indicated time points and ROS intracellular levels, measured by H2DCF-DA assay or IL-6 and IL-1β were measured by ELISA following the manufacturer’s instructions. Histograms indicate means ± SD of three diﬀerent cultures each of one was tested in triplicate. *p < 0.05, **p < 0.01, ***p < 0.001 versus unexposed cells (Ctr).
